# Tetrastatin, the NC1 Domain of the α4(IV) Collagen Chain: A Novel Potent Anti-Tumor Matrikine

**DOI:** 10.1371/journal.pone.0029587

**Published:** 2012-04-23

**Authors:** Sylvie Brassart-Pasco, Karine Sénéchal, Jessica Thevenard, Laurent Ramont, Jérome Devy, Ludivine Di Stefano, Aurélie Dupont-Deshorgue, Stéphane Brézillon, Jezabel Feru, Jean-François Jazeron, Marie-Danièle Diebold, Sylvie Ricard-Blum, François-Xavier Maquart, Jean Claude Monboisse

**Affiliations:** 1 CNRS UMR 6237, Université de Reims Champagne-Ardenne, Reims, France; 2 CHU de Reims, Laboratoire Central de Biochimie, Reims, France; 3 CHU de Reims, Laboratoire d’Anatomie et de Cytologie Pathologiques, Reims, France; 4 UMR 5086, CNRS - Université Lyon 1, IBCP, Lyon, France; Indiana University School of Medicine, United States of America

## Abstract

**Background:**

NC1 domains from α1, α2, α3 and α6(IV) collagen chains were shown to exert anti-tumor or anti-angiogenic activities, whereas the NC1 domain of the α4(IV) chain did not show such activities so far.

**Methodology/Principal Findings:**

We demonstrate in the present paper that the NC1 α4(IV) domain exerts a potent anti-tumor activity both *in vitro* and in an experimental human melanoma model *in vivo*. The overexpression of NC1 α4(IV) in human UACC-903 melanoma cells strongly inhibited their *in vitro* proliferative (–38%) and invasive (–52%) properties. MT1-MMP activation was largely decreased and its cellular distribution was modified, resulting in a loss of expression at the migration front associated with a loss of migratory phenotype. In an *in vivo* xenograft model in athymic nude mice, the subcutaneous injection of NC1 α4(IV)-overexpressing melanoma cells induced significantly smaller tumors (–80% tumor volume) than the Mock cells, due to a strong inhibition of tumor growth. Exogenously added recombinant human NC1 α4(IV) reproduced the inhibitory effects of NC1 α4(IV) overexpression in UACC-903 cells but not in dermal fibroblasts. An anti-αvβ3 integrin blocking antibody inhibited cell adhesion on recombinant human NC1 α4(IV) substratum. The involvement of αvβ3 integrin in mediating NC1 α4(IV) effect was confirmed by surface plasmon resonance (SPR) binding assays showing that recombinant human NC1 α4(IV) binds to αvβ3 integrin (K_D_ = 148±9.54 nM).

**Conclusion/Significance:**

Collectively, our results demonstrate that the NC1 α4(IV) domain, named tetrastatin, is a new endogenous anti-tumor matrikine.

## Introduction

Tumor invasion and metastasis require proteolytic degradation of extracellular matrix (ECM). This degradation involves various proteolytic cascades, such as matrix metalloproteinase (MMP) activation and the plasminogen activation system. MMPs belong to a zinc-dependent proteinase family with 23 members, secreted as inactive zymogens. MT-MMPs represent an MMP subfamily containing an additional transmembrane or anchor domain which links the enzyme to the plasma membrane. MT-MMPs, especially MT1-MMP, actively participate in the basement membrane degradation either directly or by activating latent pro-MMP-2 and pro-MMP-13 [1]–[3]. It is concentrated at the leading edge of migrating cells and interacts with caveolin-1, a caveolae component involved in endocytosis and MT1-MMP recycling to the plasma membrane [4], [5].

The extracellular matrix (ECM) is a complex structure composed of many different proteins, proteoglycans and hyaluronic acid. All basement membranes, specialized forms of extracellular matrix, comprise type IV collagen, laminins, nidogens and perlecan [6]. Six different type IV collagen α chains (α1(IV)-α6(IV)) have been identified [7]. They are composed of a 7S domain within the N-terminal domain, an interrupted triple helical domain and a globular C-terminal non-collagenous (NC1) domain [8]. The α-chains twist around one another to form a triple helix. Type IV collagen molecules associate *via* their 7S domain, their NC1 domain and laterally to form a three-dimensional network. Although the α1 and α2 chains are widely expressed and colocalize in numerous tissues, there is a temporal and spatial regulation of α3, α4, α5 and α6 expression in physiological and pathological processes.

Tumor progression and neoangiogenesis depend on a control exerted by tumor microenvironment including several intact ECM macromolecules and/or specific protein domains named matrikines [9]. Among them, the NC1 domains of several α(IV) collagen chains have been shown to inhibit angiogenesis and tumor growth [10]–[14] *via* integrin binding [15]–[20].

The NC1 α4(IV) domain was shown to lack anti-angiogenic or anti-tumor properties in a chick embryo model [10], [15]. We demonstrate here that the NC1 α4(IV) domain exerts a potent anti-tumor activity both *in vitro* and in an *in vivo* experimental human melanoma model, by decreasing proliferative and invasive properties of melanoma cells. We also provide evidence that the αvβ3 integrin could mediate the biological effects of the NC1 α4(IV) domain.

## Materials and Methods

### Ethics Statement

All animal experiments were performed in level 2 animal facilities of the Faculty of Medicine and Pharmacy of Reims, in accordance with the CNRS institutional guidelines (http://ethique.ipbs.fr/) and in conformity with the French Ministry of Research and Agriculture Charter on Animal Experimentation Ethics. Procedure of animal study was approved by the Ethics Committee of the Federative Research Institute (IFR53) from Reims Champagne-Ardenne University.

Collection and utilization of human skin biopsies were approved by the Institutional Review Board of the Reims University Hospital (CHU de Reims) and a written informed consent was required from patients.

### Reagents

Culture reagents, molecular biology products, G418 also named Geneticin (a gentamicin analog), Lipofectamine 2000 Reagent came from Invitrogen (distributed by Fischer Scientific, Illkirch, France). Bovine serum albumin, gelatin, Matrigel® (ECM gel), p3xFLAG-CMV-9 vector and anti-FLAG-M2 antibody were purchased from Sigma (St-Quentin Fallavier, France). pQE-31 vector and Ni-NTA resin were from Qiagen (Courtaboeuf, France). Goat anti-mouse MT1-MMP, anti-αvβ3 integrin antibody (23C6) were from Santa-Cruz (distributed by Tebu, Le Perray-en-Yvelines, France). The His tag monoclonal antibody was from Genscript (Piscataway, USA). Rabbit anti-human NC1 α4(IV) was produced by Covalab (Oullins, France). Ki-67 rabbit polyclonal antibody (clone EPR3610) was from Epitomics (distributed by Euromedex, Mundolsheim, France). The Alexa Fluor 488 goat anti-rabbit antibody was from Invitrogen (distributed by Fischer Scientific, Illkirch, France). Mayer’s Hemalun was from Merck (Fontenay-sous-bois, France). The anti-caveolin polyclonal antibody was from Cell signaling (distributed by Ozyme, Saint Quentin en Yvelines, France).

### Cell Culture

UACC-903 human melanoma cells were originally obtained from Dr J.M. Trent, University of Arizona Cancer Center [21]. HT-144 (HTB-63™) and SK-MEL-28 human (HTB-72™) melanoma cells were from ATCC. They were cultured in DMEM with 4.5 g/L glucose and 10% FBS at 37°C in a humid atmosphere (5% CO_2_, 95% air). Human dermal fibroblasts were isolated from skin biopsies of human donors and cultured in DMEM with 1 g/L glucose and 10% FBS at 37°C in a humid atmosphere (5% CO_2_, 95% air). In all experiments, cell viability was greater than 98%, as assessed by trypan blue exclusion.

**Figure 1:**
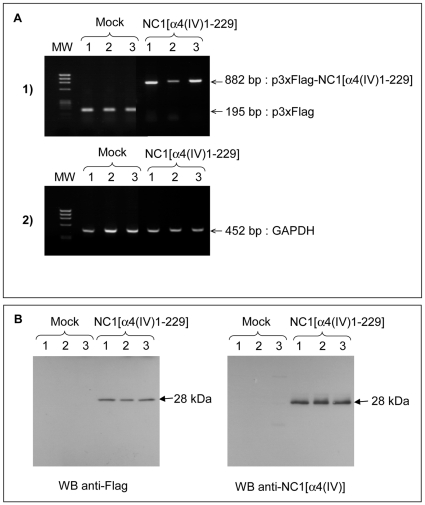
Selection of NC1 α4(IV) overexpressing cell clones. UACC-903 cells were transfected with either p3xFLAG-CMV-9 (Mock 1, 2, 3) or p3xFLAG-NC1[α4(IV)] (NC1 α4(IV) 1, 2, 3). (A): RT-PCR: Total RNA was isolated from clones selected for G418 resistance and analyzed by RT-PCR using p3xFLAG-CMV-9 (1) and GAPDH specific primers (2). (B): Western-blot: Three clones were selected by RT-PCR for their high gene expression of FLAG epitope and FLAG-NC1[α4(IV)] fusion protein. Supernatant from Mock cells and from cells stably transfected with FLAG-NC1[α4(IV)] were tested for the 28 kDa fusion protein secretion by Western blot using an anti-FLAG monoclonal antibody or an anti-NC1 α4(IV) polyclonal antibody.

### Cloning of the NC1 α4(IV) Domains in the p3xFLAG Expression Vector

*p3xFLAG-NC1α4(IV)1-229]*: The sequence encoding the complete human NC1 α4(IV) domain (NC1[α4(IV)1-229]) was amplified by reverse transcription-polymerase chain reaction from kidney mRNA using the following sets of primers: 5′-TTTGGCCCTGGATACCTCGGT-3′ and 5′-CGCATTCTCTAGCTATACTTC-3′. The resulting cDNA fragments were cloned into a p3xFLAG-CMV-9 vector. The orientation and the complete sequence of the insert were checked by sequencing. The p3xFLAG-CMV-9 expression vector contains a human cytomegalovirus promoter regulatory region that drives a high level expression while the hGH polyadenylation signal enhances mRNA longevity.

*p3xFLAG-NC1[α4(IV)180-229]*: The same experimental procedure was followed to clone the C-terminal part of the human NC1 α4(IV) domain. The sequence encoding the human NC1[α4(IV)180-229] region was amplified using the following sets of primers: 5′-CAGGGAACTTGCCACTTT-3′ and 5′-CGCATTCTCTAGCTATACTTC-3′.

### Transfection of UACC-903 Cells

The human UACC-903 melanoma cells were transfected with p3xFLAG-CMV-9, p3xFLAG-NC1[α4(IV)1-229] or p3xFLAG-NC1[α4(IV)180-229] using Lipofectamine 2000 Reagent and selected with 700 µg/mL G418. Clonal cell lines were screened by RT-PCR using fusion protein specific primers: Forward 5′-CAAAGACCATGACGGTGATTAT-3′ and Reverse 5′-TTCCAGGGCCAGGAGAGGCACT-3′ and GAPDH specific primers: Forward 5′-ACCACAGTCCATGCCATCA-3′ and Reverse: 5′-TCCACCACCCTGTTGCTGT-3′.

### Immunodetection of the FLAG-NC1[α4(IV)] Fusion Protein

To detect FLAG-NC1[α4(IV)] fusion protein by Western blot, cell supernatants were collected and proteins were precipitated with 12% trichloroacetic acid (TCA) and 0.1% Triton X-100. Proteins (1.5 µg) were separated by SDS-PAGE and transferred to an Immobilon-P^TM^ membrane. After blocking with 5% non-fat dry milk in TBS buffer (50 mM Tris, 138 mM NaCl, 2.7 mM KCl, pH 8.0), the membrane was incubated with an anti-FLAG-M2 antibody (10 µg/mL in TBS with 3% non-fat dry milk) overnight at 4°C. Then, the membrane was washed in TBS buffer and incubated with a rabbit anti-mouse IgG, peroxidase-conjugated (1/10000) for 1h30. Signals were detected using an ECL+ kit (GE Healthcare, Orsay, France), according to the manufacturer’s instructions.

**Figure 2:**
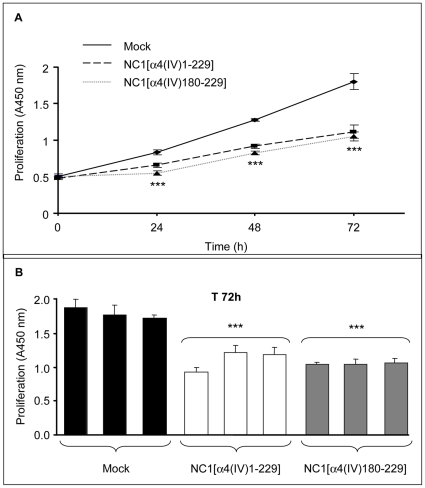
NC1 α4(IV) overexpression inhibits *in vitro* melanoma cell proliferation. (A): Cell proliferation was measured using WST-1 reagent after 24, 48 and 72 h as described in Materials and Methods (mean of three clones±SD. ***: p<0.001) (A). (B): Cell proliferation of the three selected clones at T 72 h.

### *In vitro* Proliferation Assays

Cell proliferation was evaluated using the WST-1 reagent according to the manufacturer’s instructions (Roche Diagnostic, Meylan, France). 2×10^4^ cells were seeded onto 96-well plates and incubated for 24, 48, 72 hours in 1 mL DMEM containing 2% FBS. 50 µL of WST-1 were added to the medium and, after a 20 min incubation period, absorbance was measured at 450 nm. Cell numbers were also assessed by the crystal violet assay. After the incubation periods, cells were fixed for 15 min with 1.1% glutaraldehyde, then stained for 15 min with a 0.1% crystal violet solution. Absorbance was measured at 560 nm.

**Figure 3:**
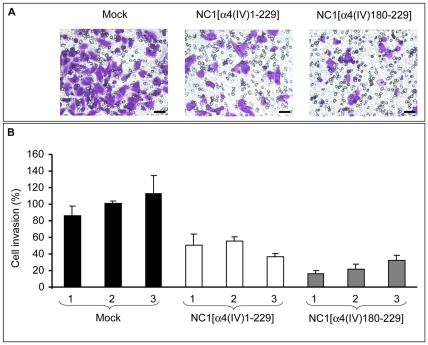
NC1 α4(IV) overexpression inhibits *in vitro* melanoma cell invasion. Mock or NC1 α4(IV) clones were tested for their ability to migrate through Matrigel®-coated membranes as described in Materials and Methods. (**A**) Representative photomicrograph shows Mock or NC1 α4(IV)-overexpressing cell invasion after a 72 h incubation period. Scale bar: 10 µm. (**B**) Representative bar graph quantifying Mock or NC1 α4(IV)-overexpressing cell invasion. ***: p<0.001.

### *In vitro* Invasion Assays

Invasion was assayed in modified Boyden chambers (tissue culture treated, 6.5 mm diameter, 8 μm pore, Greiner-One, Courtaboeuf, France). 5×10^4^ cells were suspended in serum-free DMEM with 4.5 g/L glucose containing 0.2% BSA and seeded onto membranes coated with Matrigel® (30 μg/cm^2^). The lower compartment was filled with DMEM supplemented with 10% FBS and 2% BSA. After a 72 h incubation period, cells were fixed with methanol and stained with crystal violet for 15 min. Cells remaining on the upper face of the membrane were scraped. Crystal violet staining of the migrated cells was eluted in 10% acetic acid and absorbance was read at 560 nm. The total amount of cells was also evaluated (without scraping) in each condition. The absorbance resulting from the migrating cell was reported to the absorbance of all the cells to determine the percentage of migration.

### Adhesion Assays

Cells were detached with 50 mM Hepes, 125 mM NaCl, 5 mM KCl and 1 mM EDTA, washed three times with DMEM, preincubated for 30 min with different effectors and 3×10^4^ cells were seeded per well of 96 well-plate previously coated with recombinant NC1 α4(IV) and saturated with 1% BSA. After 30, 60 or 90 min of adhesion, cells were washed three times with PBS, fixed with 1.1% glutaraldehyde and stained with crystal violet. After elution with 10% acetic acid, absorbance was read at 560 nm.

**Figure 4:**
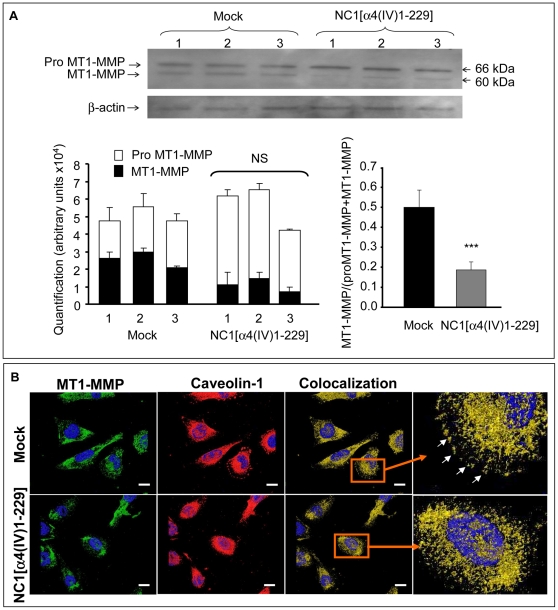
NC1 α4(IV) overexpression decreases MT1-MMP activation and modifies MT1-MMP distribution. (A): Mock or NC1 α4(IV)-overexpressing cells were incubated for 48 h without FBS. MT1-MMP expression and activation in whole cell extracts were analyzed by Western blot with an antibody directed against the hinge region of MT1-MMP. The membrane was dehybridized and reprobed with an anti-actin antibody. Quantifications were performed by densitometry using the Bio-1D software. Results were expressed as arbitrary units (AU). NS: Non significant. ***: p<0.001. (B): Cells were cultured on glass slides, fixed with paraformaldehyde and labelled with an anti-MT1-MMP directed against both the pro and the active form (green), anti-caveolin 1 (red). Yellow staining corresponds to areas where proMT1-MMP and caveolin-1 colocalized and white arrows in the insert underline the colocalization at the migration front at a higher magnification. Nuclei were conterstained with DAPI (blue). Scale bar: 5 µm.

### *In vivo* Tumor Growth Measurement

Female athymic nude mice (6 week-old; average body weight: 18–20 g) were purchased from Harlan France (Gannat, France). Animals were individually caged and given food and water *ad libitum*. They were kept in a room with constant temperature and humidity. All mice were acclimatized to laboratory conditions for 1 week before starting the experiments. A suspension of Mock or NC1 α4(IV) overexpressing-UACC-903 cells (5×10^6^ cells in 0.15 mL DMEM with 4.5 g/L glucose) was subcutaneously injected into the left side of mice. Tumor size was measured at days 10, 14, 19 and 26. Mice were sacrificed at day 26 and tumor sizes measured. Each group contained at least 10 mice. Tumor volumes were determined according to v = ½ A×B^2^, where A denotes the largest dimension of the tumor and B represents the smallest dimension [22]. Tumors were frozen in isopentane and kept at −80°C until use. 5 µm thick sections of OCT-embedded samples were fixed with 4% fresh paraformaldehyde in phosphate buffered saline (PBS), pH 7.2 at room temperature for 15 min. The slides were washed with PBS and saturated in PBS with 2% BSA, 0.1% triton X-100 in PBS for 30 min and incubated with an anti-Ki-67 rabbit monoclonal antibody (dilution: 1/100) at 4°C overnight. After washing with PBS, the slides were treated for 30 min at room temperature with the Alexa Fluor 488 goat anti-rabbit antibody (dilution: 1/200). Counterstaining was performed with Mayer’s Hemalun. Quantification was assessed using ImageJ software.

**Figure 5:**
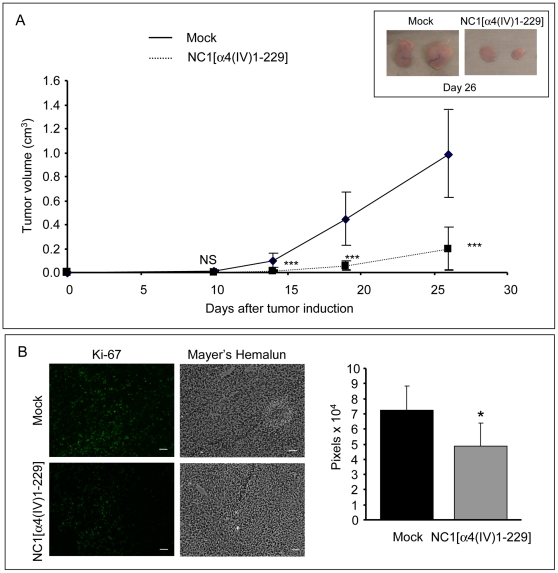
NC1 α4(IV) overexpression by melanoma cells decreases tumor growth in a mouse xenograft model. Mock or NC1 α4(IV)-overexpressing UACC-903 cells (5×10^6^ cells) were subcutaneously injected into the left side of athymic mice. (A): Tumor size was measured at days 10, 14, 19 and 26. Tumor volumes were determined according to v = ½ A×B^2^, where A denotes the largest dimension of the tumor and B represents the smallest dimension, and expressed as mean±SD (n = 10). ***: p<0.001. The insert shows example of tumors obtained in each mouse series after injection of Mock or NC1 α4(IV)-overexpressing cells. (B): Immunostaining of tumor section with an anti-Ki67 antibody. Quantification was performed with Image J. Scale bar: 50 µm.

### Western Blot Analysis

Samples were electrophoresed in a 0.1% SDS, 10% polyacrylamide gel. They were then transferred onto Immobilon-P membranes (Millipore, St Quentin en Yvelines, France). The membranes were blocked with 5% non-fat dry milk, 0.1% Tween 20 in TBS for 2 h at room temperature, incubated overnight at 4°C with anti-MT1-MMP antibody (0.2 µg/mL), and then for 1 h at room temperature with a second peroxidase-conjugated anti-IgG antibody. Immune complexes were visualized with the ECL chemiluminescence detection kit (GE Healthcare, Orsay, France).

**Figure 6:**
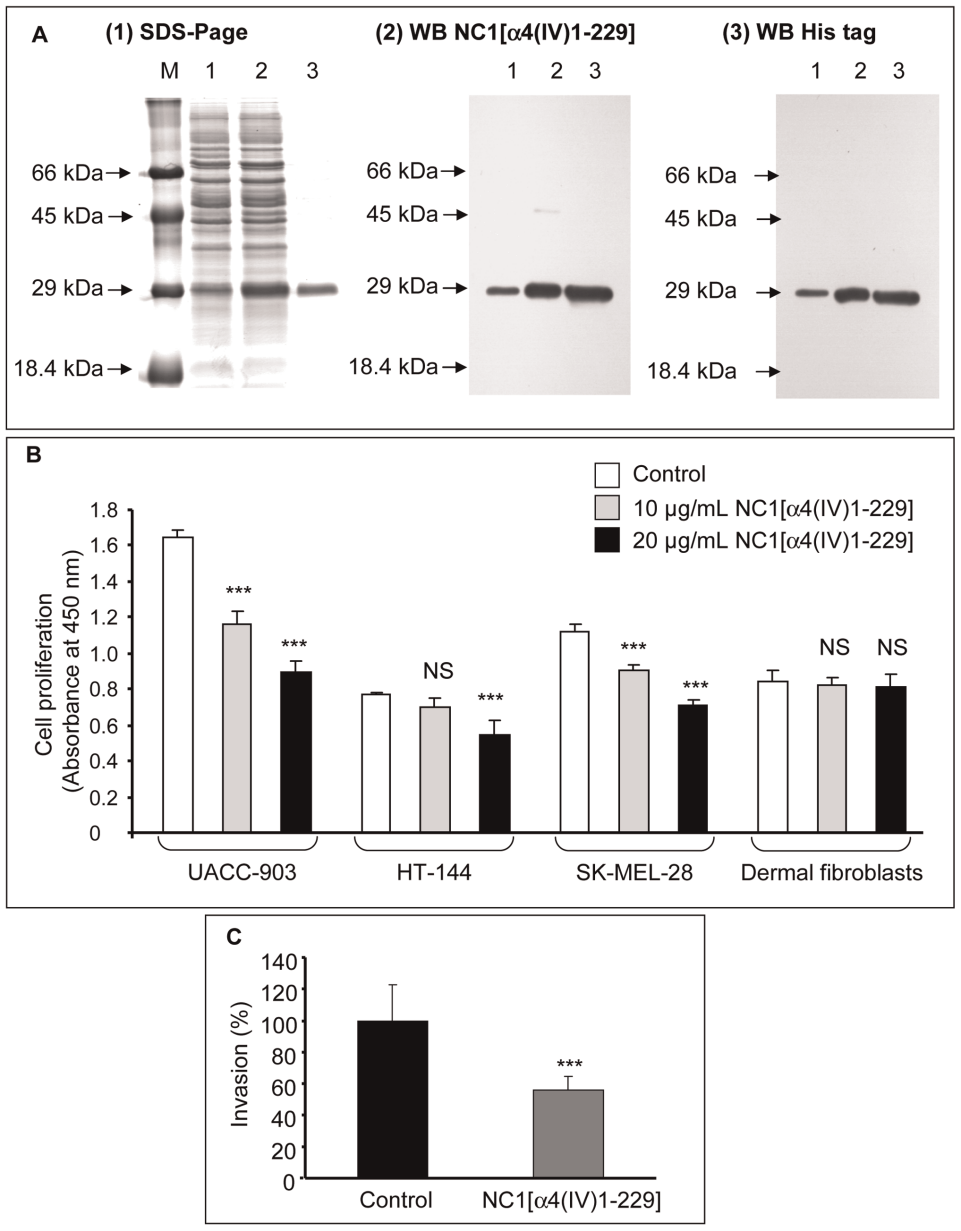
Recombinant human NC1 α4(IV) inhibits *in vitro* melanoma cell proliferation and invasion. (A): Recombinant human NC1 α4(IV) domain obtention: Recombinant human NC1 α4(IV) domain was expressed in *E. coli* JM109, DE3 strain (1) SDS-PAGE (lane M: low molecular weight markers; lane 1: T4h crude lysate, non-induced by IPTG; lane 2: T4h crude lysate, induced by IPTG, lane 3: recombinant human NC1 α4(IV) domain purified by chromatography on a Ni-NTA superflow resin. (2) Western blot using an anti-NC1 α4(IV) antibody. Lane 1, 2, 3: same as above. (3) Western blot using an anti-His tag antibody. Lane 1, 2, 3: same as above. (B): Cell proliferation: Melanoma cells and dermal fibroblasts were incubated without or with 10 or 20 µg/mL recombinant human NC1 α4(IV) for 48 h. Cell proliferation was measured as described in the Material and Methods section. NS: Non significant. ***: p<0.001. (C): Cell invasion: UACC-903 melanoma cells were incubated without or with 20 µg/mL recombinant human NC1 α4(IV). Cell invasion was measured as described in the Material and Methods section. ***: p<0.001.

### Immunofluorescence

Cells were plated on glass-slides and incubated overnight at 37°C and then fixed with 4% (v/v) paraformaldehyde for 5–10 min at 4°C. The slides were washed with PBS and saturated in PBS with 3% BSA for 30 min. Cells were then incubated overnight at 4°C with anti MT1-MMP antibody and/or with anti-caveolin-1 antibody. Slides were washed five times in PBS and cells were incubated for 1h with Alexa Fluor 488-conjugated secondary antibody to detect anti-MT1-MMP antibody and Alexa Fluor 568-conjugated secondary antibody to detect anti-caveolin-1. Both secondary antibodies were diluted 1/1000 in PBS with 3% BSA.

Cells were then washed with PBS. Control preparations were incubated without the first antibody. Nuclei were counterstained with DAPI. Immunofluorescence-labeled cell preparations were analysed using a Zeiss LSM 710 confocal laser scanning microscope with the 63X oil-immersion objective zoom 3X and Zeiss operating system (Carl Zeiss MicroImaging GmbH, Germany). Acquisitions were performed by exciting the Alexa Fluor 488, the Alexa Fluor 568 and the DAPI dye with the Argon laser, HeNe laser and chameleon IR laser tuned at 730 nm respectively. Emitted fluorescence was detected through the appropriate wavelength window. Thirty images were captured with a 0.3 μm z-step. Images were treated with Zen and AMIRA softwares.

**Figure 7:**
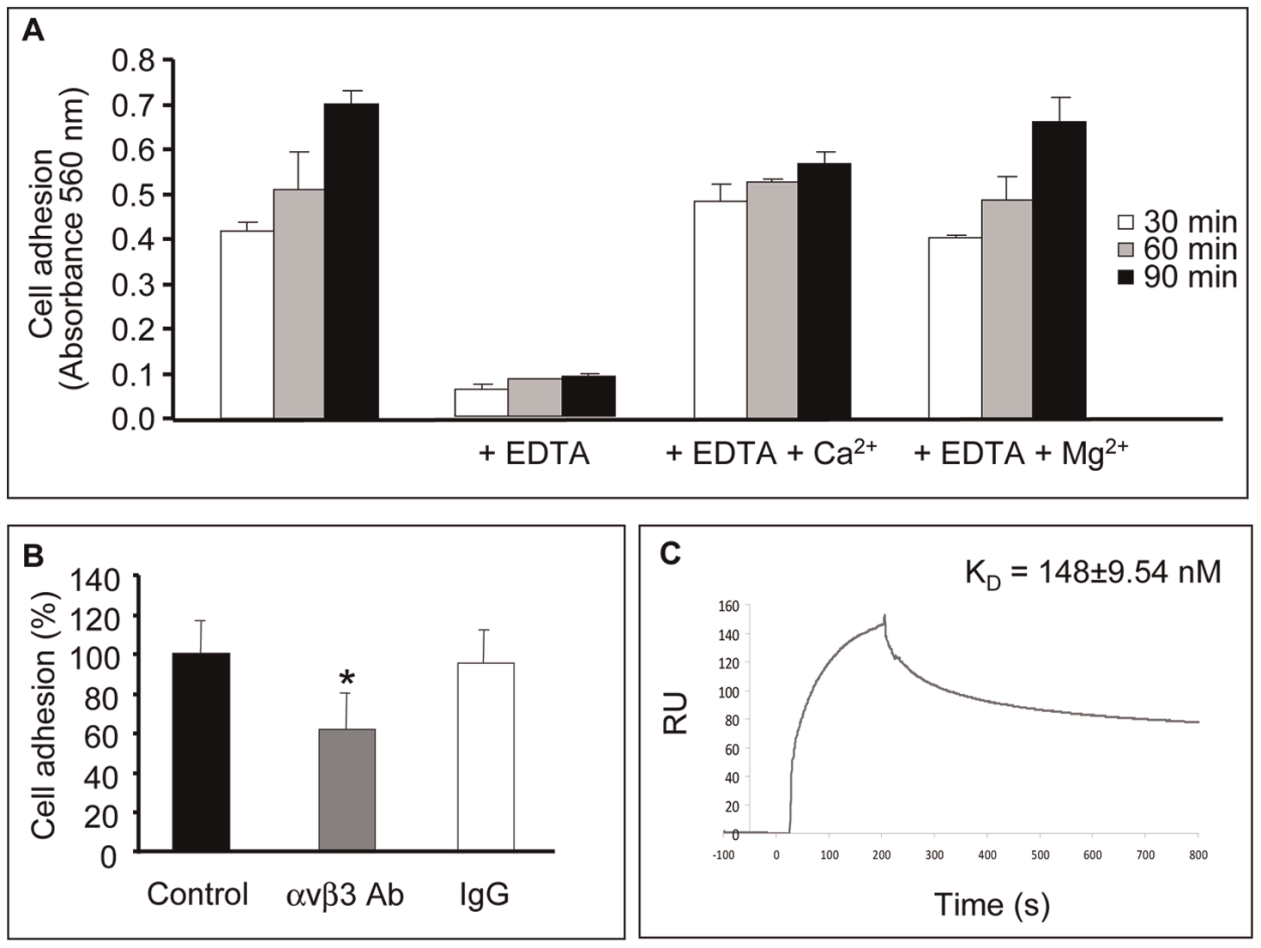
Recombinant human NC1 α4(IV) binds to αvβ3 integrin. (A): Adhesion assays were performed as described in the Material and Methods section. Cells were incubated or not with 5 mM EDTA. Cells were fixed after 30, 60 or 90 min with 1.1% glutaraldehyde and stained with crystal violet. After elution with 10% acetic acid, absorbance was read at 560 nm. *: p<0.05. Adhesion was restored by the addition of 1.3 mM Ca^2+^ or 0.5 mM Mg^2+^. (B): Adhesion assays were performed as described in the Material and Methods section. Cells were preincubated for 30 min with an anti-αvβ3 blocking antibody or an irrelevant IgG (10 µg/mL) before seeding. Cells were fixed with 1.1% glutaraldehyde and stained with crystal violet. After elution with 10% acetic acid absorbance was read at 560 nm. *: p<0.05. (C): Surface plasmon resonance (SPR) binding assays were performed by injecting recombinant human NC1 α4(IV) (3.57 µM at 30 µL/min) over αvβ3 integrin immobilized on a CM5 sensor chip (2655 RU). Binding was expressed as resonance units.

### Expression and Purification of Recombinant Human NC1 α4(IV) Domain

The sequence encoding the complete human NC1 α4(IV) domain (NC1[α4(IV)1-229]) was obtained as described in the “Cloning of the NC1 α4(IV) domains in the p3xFLAG expression vector” section.

The resulting cDNA fragments were cloned into a pQE-31 vector (Qiagen, Courtaboeuf, France). The orientation and the complete sequence of the insert were checked by sequencing. Recombinant human NC1 α4(IV) domain was expressed in *Escherichia coli* JM109, DE3 strain (Promega, Charbonnière-les-bains, France). Transformed cells were grown at 37°C in 100 mL of Luria Bertani medium containing 50 μg/mL of ampicillin until the A_600nm_ was about 0.6. Protein expression was induced by addition of 0.4 mM isopropyl-ß-D-thiogalactopyranoside (IPTG). After 4 h culture at 37°C under agitation, bacterial suspension was harvested by centrifugation at 4000 g for 15 min at 4°C. The pellets were resuspended in 5 mL of 50 mM NaH_2_PO_4_, 300 mM NaCl, 10 mM imidazole, pH 8. The cells were disrupted by thaw/freeze cycle and sonication. After centrifugation at 10 000 g for 30 min, supernatant (4 mL) was incubated for 1 h with 1 mL of Ni-NTA superflow resin. The resin was then poured into a chromatography column, washed with 4 mL of 50 mM NaH_2_PO_4_, 300 mM NaCl, 20 mM imidazole, pH 8. Finally, the recombinant domain was eluted with 3×0.5 mL of elution buffer (50 mM NaH_2_PO_4_, 300 mM NaCl, 250 mM imidazole, pH 8). After dialysis against distilled water, its purity was assessed by SDS-PAGE and by Western blotting using an anti-NC1 α4(IV) polyclonal antibody or an anti-6 His monoclonal antibody.

### Surface Plasmon Resonance Binding Assays

The surface plasmon resonance (SPR) measurements were performed on a BIAcore T100 instrument (GE Healthcare, Uppsala, Sweden, Technichal facility, IFR 128, Lyon, France). Full-length αvβ3 integrin (Millipore, Saint Quentin en Yvelines, France) was immobilized *via* its primary amine groups onto a CM5 sensor chip as previously described [23]. The immobilization level ranged from 1341 to 2655 Resonance Units (RU). Binding assays were performed at 25°C in 10 mM Hepes buffer, pH 7.4, containing 150 mM NaCl, 1 mM MgCl_2_, 2 mM MnCl_2_, and 50 mM β-octyl-D-glucopyranoside as running buffer. Recombinant human NC1 α4(IV) was injected at several concentrations and different flow rates over immobilized αvβ3 integrin. The surface was then regenerated with a pulse (60 s) of 10 mM EDTA. The kinetic parameters k_a_ and k_d_ (association and dissociation rate constants, respectively) were analyzed simultaneously using a global data analysis program (Biacore T100 evaluation software version 2.0.3) that fitted simultaneously the sensorgrams obtained at different concentrations of NC1 α4(IV), constraining the kinetic rate constants to a single value for each set of curves. R_max_, the maximal capacity of the surface was floated during the fitting procedure.

### Statistical Analyses

All the *in vitro* experiments were done in triplicate and data represent the mean of two different experiments. Statistical analyses were performed by Student’s *t* test. Results were expressed as mean±SD. For *in vivo* experiments, volumes of primary tumors were statistically analyzed using the non parametric *u*-test of Mann and Withney.

## Results

### 3xFLAG-NC1[α4(IV)1-229] Fusion Protein Production in Stably Transfected UACC-903 Cells

To assess the role of the NC1 α4(IV) domain in melanoma progression, UACC-903 cells were transfected with either the control p3xFLAG-CMV-9 or the p3xFLAG-NC1[α4(IV)1-229]. All the vectors contained the aminoglycoside phosphotransferase II gene, which confers resistance to aminoglycosides such as Geneticin (G418), allowing the selection of stable transfectants. They also encode three adjacent FLAG epitopes upstream of the multiple cloning region in which we inserted NC1[α4(IV)1-229] cDNA. This resulted in an increased detection sensitivity of the secreted FLAG-NC1[α4(IV)1-229] fusion protein. Clones selected with G418 were screened by RT-PCR for their expression of FLAG epitope (amplicon size: 195 bp) or FLAG-NC1[α4(IV)1-229] fusion protein (amplicon size: 882 bp) expression comparatively to GAPDH expression. Three clones with high FLAG mRNA expression and three clones with high FLAG-NC1[α4(IV)1-229] mRNA expression, named Mock 1, 2, 3 and NC1 α4(IV) 1, 2, 3 respectively (Figure 1A), were selected for the experiments. FLAG epitope and FLAG-NC1[α4(IV)1-232] fusion protein expressions were evidenced by immunofluorescence on Mock and NC1[α4(IV)] transfected cells respectively using an anti-FLAG antibody (data not shown). Moreover, the fusion protein (Mw: 28 kDa) was detected in the NC1 α4(IV) transfected cell supernatants by Western blot using the same anti-FLAG antibody and an anti-NC1 α4(IV) polyclonal antibody, confirming that the fusion protein was indeed secreted into the extracellular compartment (Figure 1B).

### *In vitro* Melanoma Cell Proliferation is Decreased by NC1 α4(IV) Overexpression

In order to measure *in vitro* clone proliferation, cells were seeded onto 96-well plates with 2% FBS and proliferation was measured using the WST-1 reagent after 24 h, 48 h and 72 h of incubation (Figure 2A). Figure 2B shows the data obtained with the different clones tested after 72 h. Overexpression of NC1 α4(IV) inhibited cell proliferation by 20%, 28% and 38% (p<0.001) after 24 h, 48 h, and 72 h respectively, but did not induce UACC-903 cell apoptosis in the different clones, as evaluated by Hoechst 33342 staining (Figure S1). The C-terminal NC1[α4(IV)180-229] domain also induced a strong decrease (–42%, p<0.001) in cell proliferation after 72 h (Figure 2).

### *In vitro* Melanoma Cell Matrigel® Invasion is Decreased by NC1 α4(IV) Overexpression

NC1 α4(IV)-overexpressing UACC-903 melanoma cells were tested for their ability to migrate through Matrigel®-coated (30 µg/cm^2^) filters for 72 h (Figure 3). The overexpression of NC1 α4(IV) led to a large decrease in invasion (–52%, p<0.001) comparatively to Mock tranfected cell clones. The C-terminal NC1[α4(IV)180-229] domain also induced a strong decrease (–77%, p<0.001) in cell invasion (Figure 3).

The plasminogen-plasmin activation system and matrix metalloproteinase activation are two major proteolytic cascades involved in melanoma progression [24], [25]. The overexpression of NC1 α4(IV) did not induce any significant change in the plasminogen activation system (Figure S2 A).

Matrix metalloproteinases, especially MMP-2, MMP-9 and MT1-MMP (also named MMP-14), are largely involved in extracellular matrix degradation and melanoma cell migration [25], [26]. ProMMP-9 was not detected in the conditioned media of NC1 α4(IV)-overexpressing cells and no significant change in proMMP-2 secretion was detected by gelatin-zymography analysis in the supernatant of NC1α4(IV)-overexpressing cells compared to Mock cells (Figure S2 B). Likewise, reverse zymography analysis of Tissue Inhibitors of Matrix Metalloproteinases (TIMPs) in conditioned media did not show any significant change in TIMP-2 secretion (Figure S2 C).

### NC1 α4(IV) Overexpression Inhibits MT1-MMP Activation

A crucial role of MT1-MMP has been demonstrated in cell invasion [1], [2]. NC1 α4(IV) overexpression did not modify proMT1-MMP mRNA expression measured by real time PCR analysis (data not shown). As demonstrated by Western blot using a rabbit anti-MT1-MMP polyclonal antibody that recognized both pro and active MT1-MMP, the total amount of MT1-MMP protein was not significantly altered. However, NC1 α4(IV) overexpression decreased proMT1-MMP activation by 72% as shown by measuring the active MT1-MMP/total MT1-MMP ratio (Figure 4A). Since active MT1-MMP is able to degrade extracellular matrix, the decrease in MT1-MMP activation could contribute to the inhibition of Matrigel® invasion observed with NC1 α4(IV)-overexpressing cells. Immunolocalization using the same anti-MT1-MMP antibody showed that in Mock cells, MT1-MMP was localized in the cytoplasm and at the migration front (Figure 4B). In contrast MT1-MMP was strongly decreased at the migration front in NC1 α4(IV)-overexpressing cells where it accumulated in the cytoplasm. As caveolin-1 was shown to directly interact with MT1-MMP, to promote its endocytosis and recycling to plasma membrane and therefore to increase cell migration [4], [5], we also studied caveolin-1 immunolocalization. We observed a colocalization of MT1-MMP and caveolin-1 at the migration front in Mock cells in accordance with the migratory phenotype. This aspect disappeared in NC1 α4(IV)-overexpressing cell, suggesting a loss of the migratory phenotype of the cells [insert in Figure 4B].

### *In vivo* Tumor Growth is Decreased in Mice Injected with NC1 α4(IV)-overexpressing Clones

Mock cells or NC1 α4(IV)-overexpressing cells were subcutaneously injected into the left side of athymic nude mice and tumor volume was measured at days 10, 14, 19 and 26. The clone expressing the highest level of NC1 α4(IV) was used for this experiment. Tumor volume at day 26 was decreased by 80% with NC1 α4(IV)-overexpressing cells comparatively to control Mock cells (Figure 5A). The same experiment was performed with cells overexpressing the C-terminal NC1[α4(IV)180-229] domain. No significant inhibition of tumor growth was observed in this case (data not shown).

Immunohistochemical staining of tumor sections with an anti Ki-67 antibody (Figure 5B) showed a decrease in the number of proliferating cells in tumors developed in mice injected with NC1 α4(IV)-overexpressing cells *versus* those developed in mice injected with Mock cells. This result suggests that the effect of the NC1α4(IV) domain was mediated by its antiproliferative effect *in vivo*.

### *In vitro* Effects of the Recombinant NC1 α4(IV)

To rule out a non-specific role of cell transfection in inducing a putative cell stress, we tested the effect of recombinant NC1 α4(IV) domain on the proliferation and invasion of wild type UACC-903 melanoma cells.

Recombinant NC1 α4(IV) was expressed in *E. coli* and purified or Ni-NTA resin superflow affinity column *via* its 6 His tag. Its purity was checked by SDS-PAGE and Western blot with anti-6 His and anti-α4(IV) antibodies (Figure 6A).

To assess the effects of recombinant NC1 α4(IV) on cell proliferation *in vitro*, wild type UACC-903 cells were incubated without or with recombinant NC1 α4(IV) domain (10 or 20 µg/mL). Cell proliferation was inhibited by 45% after a 48 h incubation period in the presence of 20 µg/mL of recombinant NC1 α4(IV) (Figure 6B). It also significantly inhibited the proliferation of the two melanoma cell lines HT-144 and SK-MEL-28 but not that of normal dermal fibroblasts (Figure 6B). In all conditions, cell viability was greater than 98% as determined by trypan blue exclusion.

The recombinant NC1 α4(IV) domain was also tested for its ability to inhibit tumor cell invasion (Figure 6C). Cell invasion was inhibited by 44% after a 72 h incubation period. These data suggest that post-translational modifications did not seem to be crucial for NC1 α4(IV) biological activity because the effects of the recombinant protein expressed in *E. coli* were similar to those obtained by NC1 α4(IV) overexpression in melanoma cells.

### Recombinant NC1 α4(IV) Domain Binds to αvβ3 Integrin

In order to identify the putative receptor of NC1 α4(IV) domain on melanoma cells, we studied UACC-903 cell adhesion on recombinant NC1 α4(IV). UACC-903 cells adhered to the recombinant NC1 α4(IV). Cell adhesion was inhibited by 5 mM EDTA and restored in the presence of 1.3 mM Ca^2+^ and 0.5 mM Mg^2+^ (Figure 7A). Our previous studies and other studies demonstrated that NC1 domains of α(IV) collagen chains bind to cancer cells or endothelial cells through αvβ3 integrin [16], [17], [18], [19], [20]. Therefore, UACC-903 cells were preincubated with an anti-αvβ3 integrin blocking antibody (10 µg/mL). This significantly inhibited (–38%, p<0.05) cell adhesion on recombinant NC1 α4(IV), whereas preincubation with an irrelevant IgG had no effect (Figure 7B). To confirm that αvβ3 is a receptor of NC1 α4(IV), we performed surface plasmon resonance (SPR) binding assays (Figure 7C). The NC1 α4(IV) domain bound to the immobilized αvβ3 integrin and formed a stable complex that was dissociated upon addition of 10 mM EDTA, demonstrating that the binding was dependent upon divalent cations. When the recombinant NC1 α4(IV) was injected at several concentrations over αvβ3 integrin to calculate rate constants and affinity, the experimental data were best fitted to a two-state model involving a conformational change in the first complex formed between the integrin and the NC1 domain leading to the formation of a second, more stable, complex. The calculated K_D_ = 148±9.54 nM (n = 2) showed that the NC1 α4(IV) domain bound to the αvβ3 integrin with moderate affinity.

## Discussion

Although the basement membrane provides a structural support for epithelial or endothelial cells, recent studies demonstrated that it also acts as a potential regulator of cell behaviour. During cancer progression, cross-talk occurs between tumor cells and tumor micro-environment through cell interactions with basement membrane macromolecules, mainly with its major constituent, type IV collagen. This collagen plays a pivotal role in the regulation of cell proliferation, adhesion and migration either through the full length molecule, its triple helical domain [27], [28] or through the NC1 domain of its constitutive α(IV) chains [9], [29]. For example, systemic administration of recombinant canstatin, tumstatin or NC1 α6(IV) domain strongly inhibited angiogenesis and tumor growth in various *in vivo* cancer models [15]. These matrikines inhibited endothelial cell proliferation by inducing cell apoptosis or inhibition of protein synthesis [30], [31]. Among these endogenous angiogenesis inhibitors, canstatin was also shown to exert anti-tumor properties when overexpressed by cancer cells [18]. In the same way, we previously demonstrated that tumstatin inhibited *in vitro* melanoma cell proliferation and invasion as well as tumor growth in an experimental murine melanoma model by down-regulating proteolytic cascades involved in tumor progression [13]. The anti-tumor activity of tumstatin resided in a short peptide sequence, CNYYSNS, located within its C-terminal part [32], [33]. The sequence was used as a basis to design and develop a synthetic cyclopeptide able to exert anti-tumor and anti-angiogenic activities [34], [35].

Up to now, the NC1 α4(IV) domain appeared to be devoid of anti-angiogenic activity as reported in endothelial cell adhesion and migration and in chick CAM model [10], [15]. Nevertheless, several synthetic peptides derived from type IV collagen α4 chain, named tetrastatin-1, -2 and -3 and corresponding to NC1[α4(IV)54-74], NC1[α4(IV)64-84], NC1[α4(IV)168-187] respectively, have been shown to inhibit human umbilical vein endothelial cell (HUVEC) proliferation and migration *in vitro* [36].

In the present study, we demonstrate that the NC1 α4(IV) domain has anti-tumor effects. Its overexpression by stably transfected human melanoma cells inhibited their *in vitro* proliferation without inducing apoptosis. Exogenous recombinant NC1 α4(IV) domain reproduced the anti-proliferative effect on the tested melanoma cell lines. This effect seems to be specific to tumor cells since it was not observed on dermal fibroblasts. The inhibitory effect induced by exogenously added recombinant NC1 α4(IV) domain rules out a simple induction of cellular stress due to cell transfection and/or NC1 α4(IV) domain overexpression. The NC1 α4(IV) domain also strongly decreases *in vitro* tumor cell invasion through Matrigel®-coated modified Boyden chambers. In this model, tumor cell invasion is mainly due to cell adhesion to Matrigel® and to the proteolytic degradation of Matrigel® by MMPs and the plasminogen activation system. Overexpression of the NC1 α4(IV) domain by tumor cells enhances their adhesion to Matrigel®. However, secretion of MMP-2 and MMP-9 and their endogenous inhibitors, TIMP-1 and TIMP-2, as well as plasmin activity were not affected whereas the activation of MT1-MMP was strongly decreased. Furthermore, the cellular distribution of MT1-MMP was altered with a loss of MT1-MMP at the migration front of NC1 α4(IV)-overexpressing cells suggesting a loss of their migratory phenotype. MT1-MMP is known for degrading various extracellular matrix macromolecules such as gelatin, elastin, vitronectin, dermatan sulfate proteoglycans, native fibrillar type I, II, and III collagens, and laminin-332 [37], [38]. The increase in cell adhesion and the concomitant inhibition of MT1-MMP activation and cellular distribution could explain the inhibition of the invasive properties of NC1 α4(IV)-overexpressing cells.

The NC1 α4(IV) domain inhibits *in vivo* tumor growth in a mouse xenograft model. The Ki67-immunostaining of tumor sections shows a decrease in the number of proliferating cells, contributing to the anti-tumor effect. As we previously demonstrated for tumstatin, the overexpression of the C-terminal part of the NC1 α4(IV) domain (NC1 α4(IV) 180-229) also strongly inhibits *in vitro* melanoma cell proliferation and their invasive properties, suggesting that the active sequence of the domain could be located within this domain. Surprisingly, the C-terminal fragment of NC1 α4(IV) does not have a significant effect on tumor growth in the mouse xenograft model. This intriguing result could be due to a poor secretion of the fragment by transfected tumor cells, and/or to a higher susceptibility to proteolytic degradation. Furthermore, the C-terminal fragment used in our study comprised residues 180-229. Tetrastatin-3, corresponding to residues 168-187 of the NC1 α4(IV) domain, was shown to largely inhibit HUVEC proliferation and migration [36]. It is possible that the full *in vivo* biological activity of the C-terminal fragment of NC1 α4(IV) requires several extra amino acids present in the tetrastatin-3 sequence and not in the C-terminal fragment used in our study. Further investigations to map precisely the bioactive sequence of NC1 α4(IV) are in progress.

Most collagen IV-derived matrikines act through liganding of integrins. We previously demonstrated that the anti-tumor C-terminal fragment of tumstatin binds to melanoma cell αvβ3 integrin in a RGD-independent manner [16], [39]. It was further confirmed that tumstatin also binds to endothelial cell *via* αvβ3 integrin in a RGD-independent manner [40] as well as to αvβ3 and αvβ5 integrins *via* a RGD sequence [20]. Arresten exerts also its anti-angiogenic effect on endothelial cells by liganding α1β1 integrin [41] and canstatin acts *via* αvβ3 and α3β1 integrins [18]. Our results showed that the adhesion of melanoma cells on human recombinant tetrastatin depended on divalent cations Ca^2+^ or Mg^2+^ and was abolished by EDTA and by a blocking anti-αvβ3 antibody. By surface plasmon resonance binding assays, we found that tetrastatin binds to αvβ3 integrin with a moderate affinity (∼148 nM). These results suggest that the αvβ3 integrin may serve as a receptor for tetrastatin on melanoma cells although we could not rule out the possible involvement of other integrin(s) and/or non-integrin receptor(s) in the anti-tumor activity of tetrastatin. Further studies are necessary to clarify this point.

A unique nomenclature highlights the new biological functions of several type IV collagen NC1 domains. We propose that the NC1 α4(IV) domain be named “Tetrastatin”, to highlight the molecular origin of this matrikine, the α4(IV) collagen chain and its anti-tumor activity leading to a “stasis” of *in vivo* tumor growth, as defined for Hexastatin [18]. Collagen IV-derived matrikines are promising candidates to develop new anticancer therapeutic strategies based on specific integrin targeting on both tumor cells or activated endothelial cells.

## Supporting Information

Figure S1 **NC1[α4(IV)1-229] overexpression does not induce UACC-903 cell apoptosis.** Cell nuclei were stained with Hoechst 33342 (1 μg/mL). Nuclear morphology was visualized with an inverted fluorescence microscope. No nuclear fragmentation was observed. Scale bar = 10 µm.
(TIF)

Figure S2 **Effect of NC1 [α4(IV)1-229] overexpression on proteolytic cascades.** Mock or NC1 α4(IV)-overexpressing cells were incubated for 48 h without FBS. A) Plasmin generated activity was measured in conditioned media using the H-D-Val-Leu-Lys-pNA (S-2251) peptide as a substrate and absorbance was recorded at 405 nm. NS: Non significant, ***: p<0.001. B) MMP secretion into the conditioned media was analyzed by gelatin zymography. MMP-2 quantification was performed by densitometry using the Bio-1D software. Results were expressed as arbitrary units. C) TIMP secretion into the conditioned media was analyzed by gelatin-plasminogen zymography. TIMP-2 quantification was performed by densitometry using the Bio-1D software. Results were expressed as arbitrary units.
(TIF)
