# Supplementary figures and images for: Tetrastatin, the NC1 Domain of the α4(IV) Collagen Chain: A Novel Potent Anti-Tumor Matrikine
Source: PLoS One. 2012 Apr 23;7(4):e29587. doi: 10.1371/journal.pone.0029587 (PMC3335157; doi:10.1371/journal.pone.0029587)

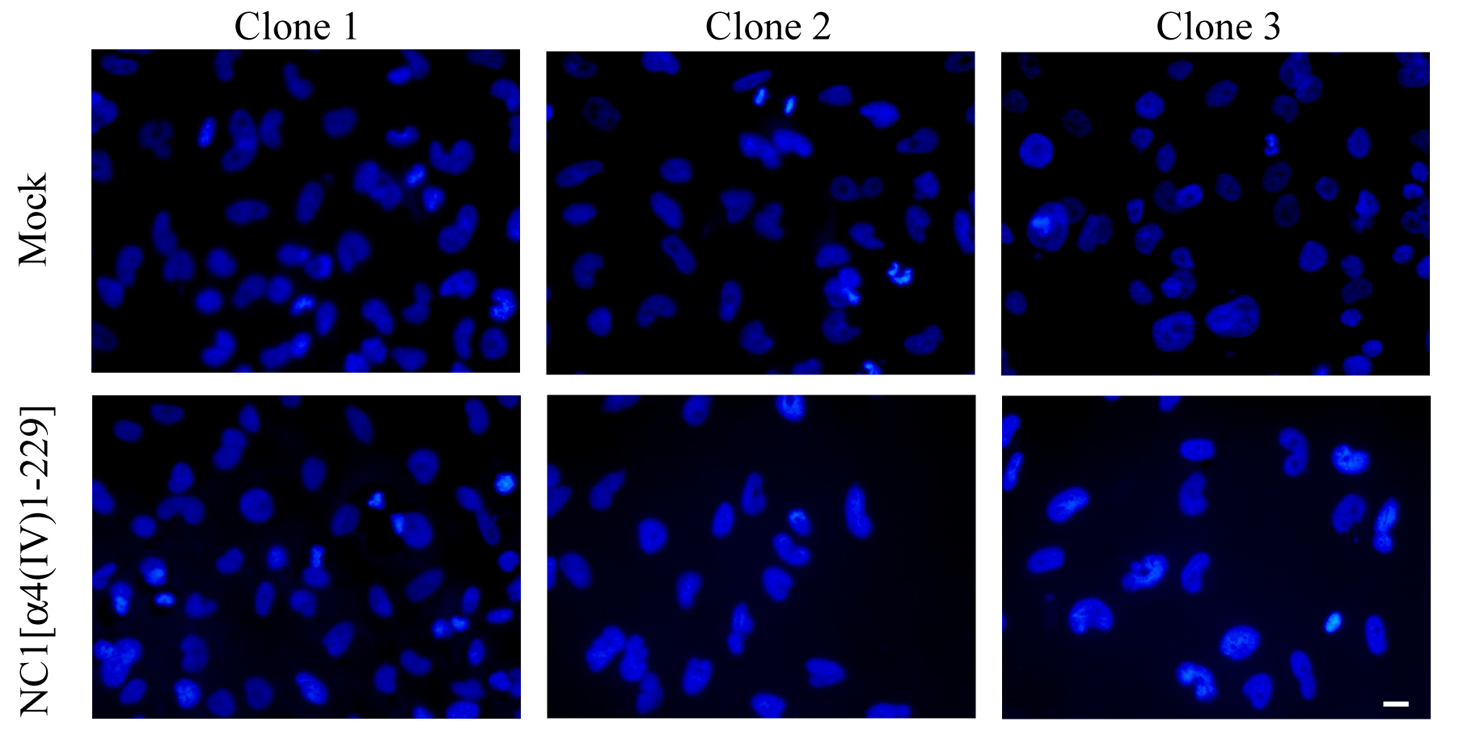

Supplement: Figure S1 — NC1[α4(IV)1-229] overexpression does not induce UACC-903 cell apoptosis. Cell nuclei were stained with Hoechst 33342 (1 μg/mL). Nuclear morphology was visualized with an inverted fluorescence microscope. No nuclear fragmentation was observed. Scale bar = 10 µm. (TIF) [file pone.0029587.s001.tif]

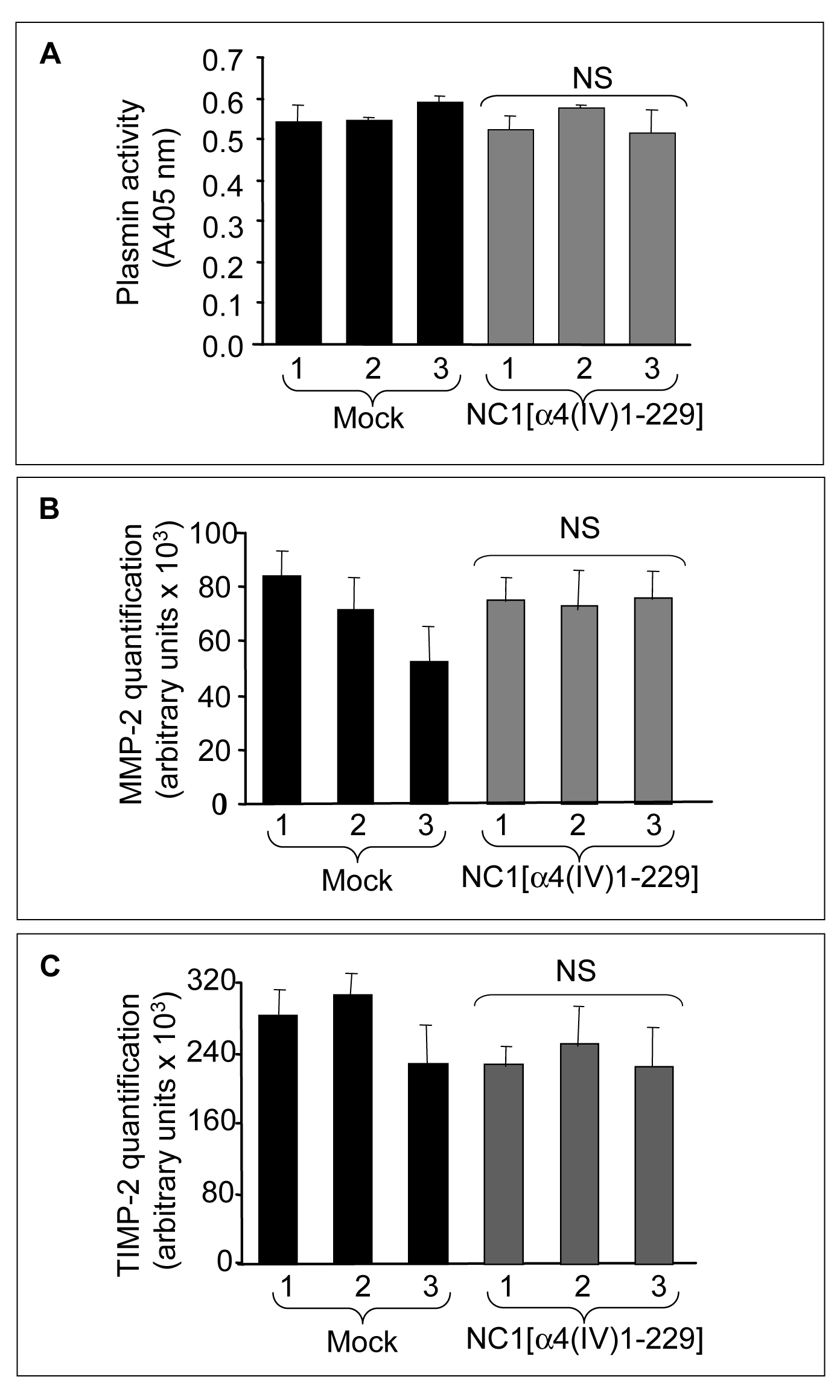

Supplement: Figure S2 — Effect of NC1 [α4(IV)1-229] overexpression on proteolytic cascades. Mock or NC1 α4(IV)-overexpressing cells were incubated for 48 h without FBS. A) Plasmin generated activity was measured in conditioned media using the H-D-Val-Leu-Lys-pNA (S-2251) peptide as a substrate and absorbance was recorded at 405 nm. NS: Non significant, ***: p<0.001. B) MMP secretion into the conditioned media was analyzed by gelatin zymography. MMP-2 quantification was performed by densitometry using the Bio-1D software. Results were expressed as arbitrary units. C) TIMP secretion into the conditioned media was analyzed by gelatin-plasminogen zymography. TIMP-2 quantification was performed by densitometry using the Bio-1D software. Results were expressed as arbitrary units. (TIF) [file pone.0029587.s002.tif]
